# Enhancing Nursing CPD, Development and Revalidation Through a Monthly Psychiatry Teaching Programme

**DOI:** 10.1192/bjo.2026.11321

**Published:** 2026-06-30

**Authors:** Michael Foster, Bethan Brace, Daniel Afloarei

**Affiliations:** North Staffordshire Combined Healthcare NHS Trust, Stoke, United Kingdom

## Abstract

**Aims:**

The aim of this initiative was to provide a structured, high-quality psychiatry teaching programme to support our Trust’s nurses’ Continuous Professional Development (CPD) and revalidation. This programme sought to further improve nurses’ clinical knowledge, prescribing skills and legal understanding.

**Methods:**

Since February 2025, monthly afternoon educational sessions were delivered face to face at Harplands Hospital, by experienced doctors, and open to all nursing staff, especially those pursuing higher education. Each session covers two clinically relevant topics, including psychosis, bipolar affective disorder, psychopharmacology, eating disorders, ADHD and medication monitoring. Attendees were encouraged to participate and to promote engagement and consolidate newly acquired knowledge, a Kahoot quiz was used each session.

Feedback was collected from attendees following each session. This questionnaire asked whether participants found the teaching helpful, whether the content was pitched at an appropriate level, asking for topic suggestions to cover in future CPD sessions, and what did they enjoy most about that day’s teaching. Open-text qualitative feedback is regularly analysed to determine quality of teaching and shape future topics.

**Results:**

Feedback has been consistently positive. The vast majority of nurses reported the sessions to be helpful and pitched at an appropriate level for their job role. Teaching was described as engaging, relevant, interactive, and supportive of their CPD and revalidation needs. Use of Kahoot and the enthusiasm of the teachers were frequently noted as being valued.

Analysis of feedback showed a recurrent theme among request including ADHD, ECG and blood test interpretation, clozapine monitoring, refeeding syndrome, mood disorders, and commoncomorbid physical health conditions. This feedback shaped subsequent teaching sessions, ensuring the programme continued to be response and learner-centred.

**Conclusion:**

The feedback from this monthly psychiatry teaching programme has successfully provided medical education to support with nursing CPD and revalidation requirements. Engagement has always been high and consistently positive feedback demonstrated the value of the programme in enhancing nurses’ knowledge, confidence, and clinical practice. Subsequently, our Trust is exploring protected teaching time for our nursing colleagues further reflecting the Trust’s commitment to continued professional development and patient care. Future plans including expanding bespoke sessions tailored to service needs and inviting novel speakers with specialist knowledge.

